# Cannabis Biomolecule Effects on Cancer Cells and Cancer Stem Cells: Cytotoxic, Anti-Proliferative, and Anti-Migratory Activities

**DOI:** 10.3390/biom12040491

**Published:** 2022-03-24

**Authors:** Hadar Peeri, Hinanit Koltai

**Affiliations:** Institute of Plant Sciences, Agriculture Research Organization, Volcani Institute, Rishon LeZion 7505101, Israel; hadarpeeri10@gmail.com

**Keywords:** cannabis, phytocannabinoids, synergy, cannabinoid receptors, cancer, cancer stem cells, cytotoxicity, glioma, glioblastoma

## Abstract

Cancer is a complex family of diseases affecting millions of people worldwide. Gliomas are primary brain tumors that account for ~80% of all malignant brain tumors. Glioblastoma multiforme (GBM) is the most common, invasive, and lethal subtype of glioma. Therapy resistance and intra-GBM tumoral heterogeneity are promoted by subpopulations of glioma stem cells (GSCs). *Cannabis sativa* produces hundreds of secondary metabolites, such as flavonoids, terpenes, and phytocannabinoids. Around 160 phytocannabinoids have been identified in *C. sativa*. Cannabis is commonly used to treat various medical conditions, and it is used in the palliative care of cancer patients. The anti-cancer properties of cannabis compounds include cytotoxic, anti-proliferative, and anti-migratory activities on cancer cells and cancer stem cells. The endocannabinoids system is widely distributed in the body, and its dysregulation is associated with different diseases, including various types of cancer. Anti-cancer activities of phytocannabinoids are mediated in glioma cells, at least partially, by the endocannabinoid receptors, triggering various cellular signaling pathways, including the endoplasmic reticulum (ER) stress pathway. Specific combinations of multiple phytocannabinoids act synergistically against cancer cells and may trigger different anti-cancer signaling pathways. Yet, due to scarcity of clinical trials, there remains no solid basis for the anti-cancer therapeutic potential of cannabis compounds.

## 1. Introduction

Cancer is a complex family of diseases, in which a gradual change in the expression of multiple genes leads to genomic instability and cell death imbalance, resulting in the abnormal growth of cells [1]. Although different types of cancer present with different phenotypic clinical characteristics and different genetic modifications, there are several common molecular patterns and biological capabilities acquired during malignant transformation. The hallmarks of cancer comprise six distinctive and complementary processes essential for tumor growth and survival: sustaining proliferative signaling insensitivity to growth suppressors; disproportionately greater growth over cell death; limitless replicative potential; and the induction of angiogenesis, tissue invasion, and metastasis [2].

*Cannabis sativa* L. (*C. sativa*) is a diecious annual herb belonging to the Cannabaceae family and has been effective in treating numerous medical conditions [3,4]. The major utilization of cannabis is for recreational purposes. While many countries are legalizing cannabis production and use, cannabis remains the most widely used illegal drug globally [5]. However, the medical use of this plant has been documented in the oldest Chinese pharmacopoeia pen-ts’ao ching (compiled in 100 CE but attributed to Emperor Sheng Nung, c. 2700 BCE) for pain relief, constipation, and other ailments. In India, the plant was historically used for analgesic, tranquilizing, anesthetic, antibiotic, and anti-inflammatory functions [6,7,8]. Around 600 constituents have been identified in *C. sativa*, among them being several classes of secondary metabolites, including dozens of flavonoids, hundreds of terpenes, and more than 160 terpenophenolic compounds known as phytocannabinoids [9,10,11,12]. Among the most abundant phytocannabinoids are Δ9-tetrahydrocannabinol (THC), cannabidiol (CBD), and cannabigerol (CBG), which are all synthesized by female plants and stored mainly in epidermal glandular trichomes, which are densely concentrated in the inflorescence and bracts. Phytocannabinoids are produced as prenylated aromatic carboxylic acids and converted to neutral homologous forms by decarboxylation, which occurs to some extent within the living plant but mostly when catalyzed by heat following harvesting [9,10,11,12]. Today, several cannabis preparations or synthetic compounds have been approved by health authorities worldwide (e.g., FDA or EU) and meet the same regulatory requirements of pharmaceutical drugs in terms of safety, efficacy, and consistency. These include Nabiximols, which is a whole-plant prescription cannabinoid used in the management of patients with multiple sclerosis, chronic neuropathic pain, and cancer-related pain [13]. Another example is Dronabinol, a synthetic phytocannabinoid (THC) that is marketed as medicines in several countries and which is indicated for the treatment of anorexia and weight loss in adult patients with HIV/AIDS or cancer [14].

## 2. The Nature of Stem Cells

There is evidence that malignant solid tumors contain a subpopulation of cancer stem cells (CSCs) that have a clonogenic and tumorigenic potential. Similar to stem cells, CSCs are characterized by a capacity for self-renewal, in which one cell generates more stem cells. CSCs also possess an ability for multi-lineage differentiation, which increases genetic heterogeneity within the tumor mass [15,16]. Importantly, CSC may not be considered as a discrete entity. Rather, CSC plasticity was identified as a range of attributes in a CSC state. Stem-to-nonstem and nonstem-to-stem transitions in daughter cells take place in various cancers, in a “bidirectional interconversion” mode [17]. Moreover, CSCs in various cancer types are influenced by neighboring cancer cells to create a perivascular niche, and they are affected by the tumor microenvironment [17]. CSCs are highly enriched in the stemness pathways. In the case of triple-negative breast cancer stem cells, the involvement of Notch, JAK-STAT, Wnt/β-catenin, and Hedgehog pathways was demonstrated. These signaling pathways are known to play an important role in the proliferation and differentiation of cancer stemness [18]. In accordance, a number of cell surface markers, such as the clusters of differentiation (CD) markers CD24, CD133, CD44 and aldehyde dehydrogenase 1 (ALDH1), were shown to be associated with CSCs in various cancers [19,20]. These proteins are mostly “functional markers” and may act as mediators of one or another aspect of stem behavior. Matching a stem marker with a stem behavior is at an early stage but an important unmet goal.

CSCs have been implicated in tumor initiation and infiltration as well as tumor progression and recurrence. Furthermore, it was found that CSCs stimulate tumor angiogenesis and invasion and are major drivers of metastasis and tumor resistance [15,16]. CSCs also show resistance to conventional cancer therapies such as radio- or chemotherapy [16].

Notably, the non-stem subpopulation also plays an important role in a malignant tumor’s growth, as a non-stem population may reconstitute a stem population [17]. Nevertheless, identifying novel cancer treatments that target CSCs is of great importance.

## 3. Glioma

One of the most complicated and treatment-resistant cancers is glioma. Gliomas account for ~80% of all malignant brain tumors [21]. Gliomas are classified from grade I (benign) to IV (malignant), according to the World Health Organization (WHO) classification. They are also classified according to molecular factors that define tumor entities. Glioblastoma multiforme (GBM, WHO grade IV glioma), the most common subtype of brain tumor, is an extremely invasive, aggressive, and lethal type of cancer, with poor prognoses [21].

Despite aggressive multidisciplinary treatments, the median survival rate for patients diagnosed with GBM is under two years from diagnosis and has shown no significant improvement in decades [22]. Standard GBM therapy approaches include maximal surgical resectioning followed by radio- and chemotherapy [22]. However, no standard of care has been established in recurrent or progressive GBM, and treatments include surgery, re-irradiation, systemic therapies, combined modality therapy, and supportive care [23].

In general, GBM often display a genetic and microscopic structural heterogeneity and significant pathology within the tumor mass due to the presence of different subpopulations of cells, including glioma stem cells (GSCs). GSCs are a minor population of pluripotent and self-renewing cancer cells [24,25]. GSCs maintain unlimited proliferation and thereby support tumor growth and recurrences. Tumor rapid growth is dependent on the GSCs progenitor cells that are fast dividing; tumor recurrences often result from the low mitotic activity of GSCs. This low mitotic activity protects them from the various treatments that actively target dividing cells. As a result, GSCs can survive these treatments and give rise to recurrences [25]. Consequently, effective therapies that target both GBM cells and GSCs are urgently needed to improve the prognosis and quality of life for GBM patients.

## 4. Anti-Cancer Properties of Cannabis Compounds

### 4.1. Pre-Clinical Studies

Studies have demonstrated that phytocannabinoids potentially possess anti-cancer properties, including the inhibition of cell migration, proliferation, and angiogenesis and the induction of apoptosis in skin, lung, breast, prostate, and glioma cancer cells [26,27,28,29]. Phytocannabinoids trigger cancer cell death via various signal transduction pathways, including oxidative stress, cell cycle arrest, endoplasmic reticulum (ER) stress, autophagy, and apoptosis [26,27,28,29].

One of the most abundant phytocannabinoids, THC, was shown to inhibit the growth of some tumors, inhibit angiogenesis, and induce apoptosis in various cancers cells in vitro and in vivo [27,28,29,30,31]. THC and CBD exhibited synergistic inhibition of cell proliferation in GBM cell lines [32]. Furthermore, CBD was found to inhibit the invasiveness of breast cancer cells and GBM cells at sub-lethal concentrations by downregulating matrix metalloproteinases (MMPs) and their inhibitors (TIMPs) [33,34]. An MMP–TIMP imbalance results in proteolysis of the matrix that may be associated with different pathological processes, including tumor invasion [35]. In vivo, THC and/or CBD reduced GBM tumor growth [36]. Furthermore, several studies have demonstrated CBG anticancer activity, including in mouse melanomas, human oral epithelioid carcinoma cells, human breast carcinomas, and colorectal cancer cells [28].

Recently, we have shown that two fractions of a high-THC cannabis strain extract had a significant cytotoxic activity against Human GBM cell lines and GSCs derived from Human tumor specimens [37]. The two fractions were composed of different combinations of phytocannabinoids, with CBG or THC as the most abundant compound. The active fractions induced apoptosis and the expression of ER-stress-associated genes. Moreover, the fractions altered cell cytoskeletons, reduced cell invasion, and inhibited cell migration and colony formation [37]. Notably, the study demonstrated the therapeutic potential of combinations of cannabis compounds in exerting cytotoxic, anti-proliferative, and anti-migratory effects on human GBM cells. Furthermore, the activity of these specific combinations was higher than that of the purified primary compound in each fraction, as well as that of the crude extract [37]. Notably, in many cases, phytocannabinoid concentrations used in vitro do not coincide with those safely achievable in vivo, and clinical trials are needed to prove phytocannabinoid treatments’ efficacy.

### 4.2. A Clinical Study

One promising clinical evidence suggests effective phytocannabinoid-based treatments against GBM [38]. A pilot phase I clinical trial indicated that THC has a good safety profile [39]. The administration of THC in two of nine GBM patients in this trial led to a decrease in tumor cell proliferation [39].

## 5. Entourage and Synergies between Cannabis Compounds

Many studies have suggested that the natural combinations produced by the plant are more effective than treatments with a single compound, owing to what has been termed the ‘entourage effect’ [40,41]. Two sub-types of the entourage effect are known: ‘intra-entourage’, which refers to the enhancement of the biological activity by the interaction of different phytocannabinoids, and ‘inter-entourage’, which refers to the enhancement of the biological activity by the interaction of phytocannabinoids and other cannabis secondary metabolites, such as terpenes [41].

Several studies have demonstrated the intra-entourage effect between phytocannabinoids. For example, the synergistic interaction between THC and cannabichromene (CBC) was identified in a study on human bladder urothelial carcinoma (UC) cells, the most common urinary system cancer. The synergistic combination led to cell cycle arrest and apoptosis, altered cytoskeleton organization, and inhibited cell migration [42]. In another example, a study on human cutaneous T-cell lymphoma (CTCL) cells found that a combination of phytocannabinoids that contained CBD, CBG, THC, and CBC was more cytotoxic to the cells than CBD, the primary compound, solely. In addition, the treatment led to apoptotic cell death and induced the expression of ER-stress-related genes [43]. More evidence was shown in a study on colorectal cancer cell lines and colon polyps, where the synergistic interaction of a cannabigerolic acid (CBGA)-rich fraction and a Δ9-tetrahydrocannabinolic acid (THCA)-rich fraction resulted in a reduction of the IC50 values compared to each fraction alone [44]. In addition, the synergistic combination induced apoptotic cell death, increased G0/G1 cell cycle arrest, and led to differentially expressed genes, including genes involved in the p53 and Wnt signaling pathways, compared to gene expression following treatment with each fraction separately [44].

Additional studies on leukemia and multiple myeloma (MM) cells found that the combination of THC and CBD was more effective than each compound on its own. In leukemic cells, when THC and CBD were combined at a 1:1 ratio, the IC50 value of the combination was two-fold lower and approximately three-fold lower compared to CBD IC50 and THC IC50, respectively [45]. In MM cells, the combination showed higher activity in inducing cell cycle arrest and autophagic cell death [46].

The mechanism behind the entourage or synergistic effect could be explained by the activation of multiple receptors by phytocannabinoids (detailed below). When different phytomolecules activate more than one receptor, intensified anti-tumor activity may be expected. Alternatively, the activation of several signaling pathways by phytocannabinoids in parallel may lead to synergistic activity [41].

## 6. Activity of the Endocannabinoid System Is Altered in Numerous Types of Cancer

The endocannabinoid system (ECS) is a signaling network that consists of cannabinoid receptors, endogenous ligands (termed endocannabinoids), and metabolic enzymes [47]. The ECS is widely distributed in the body, and it has an important role in maintaining a homeostatic balance and in the regulation of various physiological processes, such as synaptic transmission and immunomodulation [47]. Dysregulation of the ECS is associated with different diseases, including obesity, diabetes, anxiety and depression, inflammation, neurodegenerative disorders, multiple sclerosis, schizophrenia, glaucoma, cardiovascular diseases, obesity, and cancer [48,49]. Similarly, ECS activity is altered in numerous types of cancer [26,27], and its modulation has been suggested to have therapeutic effects on a wide range of pathological conditions [48] and even to be a target for cancer treatment [49,50].

## 7. Cannabinoid Receptors and Their Activation

Cannabinoid receptors can be activated by interaction with endo-, phyto- or synthetic cannabinoids. The cannabinoid receptors type 1 and type 2 (CB1 and CB2) belong to the seven-transmembrane G-protein coupled receptor (GPCR) superfamily and are among the most abundant subtype in the body [51]. In addition, there are other GCPRs and ion channels that can be activated by interaction with cannabinoids, such as G-protein coupled receptor 55 (GPR55), the transient receptor potential vanilloid (TRPV) family, TRP ankyrin (TRPA) family, and peroxisome proliferator-activated receptors (PPARs), among others [52,53].

THC acts as an agonist (activator) of both CB1 and CB2 receptors. CB1 activation by THC is associated with hypothermia, catalepsy, the suppression of locomotor activity, desensitization of pain, and appetite enhancement. Activation of CB2 by THC is associated with anti-inflammatory effects and pain relief [12]. CBD may act as a CB1 antagonist and, particularly in the presence of THC, may counteract some of the unwanted side effects of THC, including intoxication, increased appetite, anxiety, tachycardia, and sedation. CBD is also an agonist for TRPV1 and 5-HT1A receptors, having anti-inflammatory, anticonvulsive, and anti-psychotic effects [12]. Although the activation of cannabinoid receptors has been shown to inhibit tumor progression [49], there is still a lack of understanding of the mechanisms through which cannabinoids receptors produce anti-tumor processes. For example, CB2 receptor expression was found to positively correlate with the tumor malignancy grade in GBM cell lines and tissue biopsies compared to normal tissues, which express mostly CB1 receptors [36]. CB1 and CB2 receptors, as well as other elements of the ECS, have been found to be expressed in GSC derived from GBM biopsies [54]. However, there are inconsistent data about CB1 receptor expression in GBM cells [36]. Treatments with selective CB2 antagonists prevented glioma tumor regression induced by its agonist in vivo [55] and phytocannabinoids’ cytotoxicity and expression of ER-related genes [37].

## 8. Intracellular Effects of Phytocannabinoids in Glioma Cells

**Figure 1:**
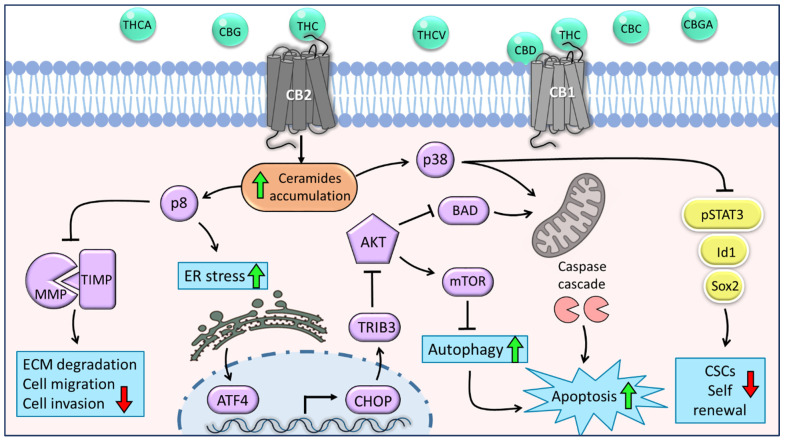
The main molecular mechanisms underlying the anti-tumor effects of *C. sativa* phytocannabinoids on glioma cells and glioblastoma stem cells. Phytocannabinoids inhibit cell viability and motility through various cannabinoid receptor (CB)-mediated mechanisms. THC acts as an agonist of both CB1 and CB2 receptors; CBD may act as a CB1 antagonist. The activation of CB1 or CB2 stimulates the synthesis and accumulation of ceramides (orange shape) and, as a result, triggers the induction of p8. This leads to the inhibition of cell migration and invasion through the downregulation of MMPs. Furthermore, p8 promotes the upregulation of ER-stress-related genes *ATF-4*, *CHOP*, and *TRIB-3*, followed by inhibition of the Akt-mTORC1 axis and initiation of autophagy, which is upstream of apoptosis. In addition, inhibition of Akt leads to the overexpression of BAD and consequently induces apoptosis via the intrinsic mitochondrial pathway. Another signaling pathway activated by ceramides is p38-MAPK, which involves both apoptosis activation and inhibition of CSC self-renewal through the downregulation of stemness regulators, such as p-STAT3, Id1, and Sox2 (yellow shapes). Green arrows represent upregulation and red arrows represent downregulation of biological processes. Purple shapes represent genes or proteins, and blue shapes represent biological processes.

Considering the complexity and the wide distribution of ECS components and their interaction with phytocannabinoids [47,49], phytocannabinoids may have the potential to impact and mediate a multitude of cancer-related signaling pathways. One common pathway activated by phytocannabinoids in different cancer types is the ER-stress pathway, which is one of the main mechanisms to induce apoptosis of glioma, astrocytoma, melanoma, and pancreatic tumor cells [56]. Previous studies on several models of glioma reported that CB1 receptor agonists and, more efficiently, CB2 receptor agonists stimulated the synthesis and accumulation of ceramide, a pro-apoptotic lipid second messenger which leads to the induction of stress protein p8 ([31,57]; Figure 1). Following this p8 induction, downstream ER-stress-related genes were induced (Figure 1), and as a result, the intrinsic mitochondrial pathway was activated [31,57].

Recently, we have shown that CBG-rich and THC-rich combinations of phytocannabinoids induced Activating transcription factor 4 (*ATF4*), C/EBP homologous protein (*CHOP*)-*10* (GADD153/DDIT-3), and Tribbles homolog 3 (*TRIB3*) gene transcription in a CB2 activation-dependent manner ([37]; Figure 1), supporting the notion that phytocannabinoid treatments induce cell death via ER stress. ATF4 is a transcription factor transiently induced following treatment with ER stressors [56]. In turn, ATF4 induces *CHOP* expression, a transcription factor that regulates the expression of many pro- and anti-apoptotic genes [58]. Under ER-stress, CHOP activates pro-apoptotic proteins, including the B cell lymphoma-2 (BCL-2) family proteins, such as BAK and BAX, and represses anti-apoptotic BCL-2 family proteins [58]. TRIB3 is a pseudokinase and another protein associated with ER-stress, which was found to facilitate ER-stress-dependent apoptosis via the NF-κB pathway [59]. Moreover, TRIB3 has been shown to inhibit the Akt-mTORC1 axis, consequently leading to the initiation of autophagy (Figure 1), which is upstream of intrinsic mitochondrial apoptosis [60].

Furthermore, treatment with the cannabinoid-receptor synthetic agonist WIN-55,212-2 led to upregulation of the BCL-2 homology 3 (BH3)-only family member BAD, a pro-apoptotic protein, in response to ceramide activation and the serine/threonine kinase Akt downregulation in glioma cells ([61]; Figure 1). Ceramide is also an important regulator of p38 mitogen-activated protein kinase (MAPK), and previous studies on human leukemia and glioma cells reported that following THC treatment, activation of this pathway induced apoptosis partially via the CB1 and CB2 receptors ([57,62]; Figure 1).

Importantly, in contrast to malignant cells, normal brain cells, such as primary neurons and astrocytes, do not undergo apoptosis or present ceramide accumulation in response to phytocannabinoid treatments [31]. In addition, it has been shown in vivo that even at high doses, there is no sign of any damage or neurotoxicity to normal brain tissue following treatments with phytocannabinoids [63]. These findings, together with the differences in the expression of cannabinoid receptors between normal tissues and cancer cells (detailed above), and the fact that cannabinoid receptors mediate the anti-cancer activities support the suggestion that cannabinoid receptors regulate cell survival and cell death signaling pathways differently in glioma cells and non-transformed cell [64].

Although the role of cannabis compounds in the suppression of cancer migration and invasion is elusive and poorly characterized, accumulating evidence suggests that cannabis compounds have potent anti-migrative and anti-invasive effects on GBM cells, both in vitro and in vivo. It was previously reported that treatment with THC or CBD down-regulated the expression of major proteins associated with glioma tumor migration, in particular MMP-2, MMP-9, TIMP-4, and TIMP-1 [34,65], even at low concentrations, which were insufficient to induce cell apoptosis. TIMP-1 and some MMP expression is selectively upregulated in different cancers and strictly associated with tumor malignancy and metastasis [66]. Interestingly, THC treatment depressed TIMP-1 and MMP-2 expression in glioma cell lines as well as in cultured human GBM primary cells. In addition, the local administration of THC down-regulated TIMP-1 and MMP-2 expression in glioma-bearing mice and in two patients with recurrent GBM [30,65]. Moreover, these effects of THC were suggested to be mediated via CB2 receptor activation and were prevented by the blockade of ceramide synthesis and by knock-down of the p8 stress protein in glioma cells ([65]; Figure 1).

## 9. Phytocannabinoids’ Activity against Glioblastoma Stem Cells in Pre-Clinical Studies

The CSC hypothesis, which suggests that a small subset of stem cells is responsible for tumor initiation, progression, and drug resistance [16], prompted extensive research on CSCs. The presence of GCSs in high-grade gliomas is well-established, and it has been suggested that the existence rates of these cells increase proportionally with the grade of gliomas [67].

It has been demonstrated previously that GBM tumors and cell lines contain a subpopulation of cells that can form tumor-neurospheres [68]. These neurospheres are enriched with cells that share stem cell characteristics such as multipotency, self-renewal, and generation of secondary spheres. Moreover, the implementation of cells isolated from GBM neurospheres was able to form tumors in-vivo [69]. Altogether, this subpopulation of cells may represent GSCs to some extent.

It was demonstrated that the activation of cannabinoid receptors alters the expression of regulatory genes associated with stem cell proliferation and differentiation and inhibits the invasiveness and tumorigenesis of GSCs [54]. Similarly, we have shown that specific combinations of phytocannabinoids have the potential to target key signaling pathways affecting GSCs’ viability and motility. THC-rich or CBG-rich phytocannabinoid combinations had significant cytotoxic activity against GSCs from a GBM primary tumor [37]. Moreover, treatment of GSCs with the active combinations at sub-lethal concentrations inhibited neurosphere formation in 2- and 3-dimensional models. Hence, these cannabis treatments may have the potential to prevent the formation of GBM neurospheres [37].

CBD was shown to increase reactive oxygen species (ROS) and activate the p38-MAPK signaling pathway, which led to the inhibition of cultured primary GSC survival and self-renewal, and the downregulation of key stem cell regulators, such as Inhibitor of DNA binding 1 (Id1), Sox2, and p-STAT3 ([70]; Figure 1). Furthermore, CBD treatment stimulated the activation of caspase-3 in GBM in vivo and prolonged the survival of mice bearing intracranial GBM xenografts derived from GSCs [70].

Previously, it was reported that GSCs isolated from GBM biopsies and human glioma cell lines express cannabinoid receptors, in particular, CB2, and other ECS elements, including the enzymes responsible for endocannabinoid degradation, MAGL and FAAH [54]. In this study, the activation of CB receptors by synthetic cannabinoid agonists down-regulated genes involved in cell cycle progression and cell proliferation and increased the transcription levels of the tumor suppressor RBL1. Moreover, the synthetic cannabinoid agonists promoted GSC differentiation that damaged the cells’ ability to initiate glioma generation and tumor growth in vivo [54].

## 10. Summary and Concept

Despite numerous findings regarding the cytotoxic effects of phytocannabinoids on various cancers in cell cultures and animal models, GBM included, there remains no solid basis for the therapeutic potential of cannabis compounds due to the scarcity of clinical trials.

**Figure 2:**
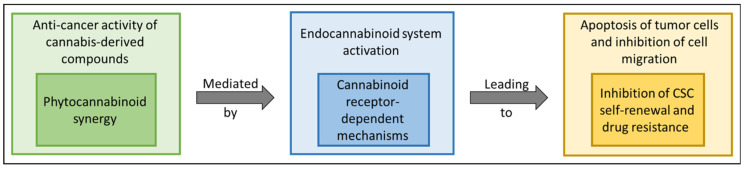
Conceptual perspective of the anti-cancer activity of phytocannabinoids. Cannabis compounds and phytocannabinoids, in particular, by activating cannabinoid receptor-dependent mechanisms, may interact synergistically in some of the cases and target malignant cells by inducing, e.g., cell apoptosis and inhibition of cancer cell migration. Moreover, phytocannabinoids may target CSCs, in some cases leading to an improved outcome, e.g., by inhibiting the characteristic self-renewal and drug resistance of CSCs.

Among others, the studies summarized here suggest that cannabinoids may target malignant cells and CSCs by activating cannabinoid receptor-dependent mechanisms (Figure 2) and could be useful as an adjuvant therapy to complement and improve the current standard of care. The anti-tumor action of the ECS is well-established in models of various cancers; however, its involvement against CSCs remains to be well characterized and should be further examined.

## Data Availability

Not applicable.

## Abbreviations

ATF4	Activating transcription factor 4
BCL-2	B cell lymphoma 2
BH3	BCL-2 homology 3
CB1/2	Cannabinoid receptor type 1/type 2
CBC	Cannabichromene
CBD	Cannabidiol
CBG	Cannabigerol
CBGA	Cannabigerolic acid
CBN	Cannabinol
CHOP	C/EBP homologous protein
CNS	Central nervous system
CSCs	Cancer stem cells
CTCL	Cutaneous T-cell lymphoma
ECS	Endocannabinoid system
ER	Endoplasmic reticulum
GBM	Glioblastoma multiforme
GPCR	G-protein coupled receptor
GSCs	Glioma stem cells
IC50	Half maximal inhibitory concentration
Id1	Inhibitor of DNA binding 1
MAPK	Mitogen-activated protein kinase
MM	Multiple myeloma
MMP	Matrix metalloproteinases
MTORC1	Mammalian target of rapamycin complex 1
PPARs	Peroxisome proliferator-activated receptors
ROS	Reactive oxygen species
THC	Δ^9^–tetrahydrocannabinol
THCA	Δ^9^–tetrahydrocannabinolic acid
THCV	Δ^9^–tetrahydrocannabivarin
TIMP	Tissue inhibitor of metalloproteinases
TMZ	Temozolomide
TRIB3	Tribbles homolog 3
TRPA	Transient receptor potential ankyrin
TRPV	Transient receptor potential vanilloid
UC	Urothelial carcinoma
WHO	World Health Organization
